# ‘Sometimes sheep need a vet’: A qualitative study of Pentecostal clergy knowledge, attitudes, beliefs, and behaviours regarding mental health

**DOI:** 10.1371/journal.pone.0342407

**Published:** 2026-03-11

**Authors:** Justin Muthaih, Adam Caplin, G. Eric Jarvis, Rob Whitley

**Affiliations:** 1 Department of Psychiatry, Douglas Mental Health University Institute, McGill University, Montreal, Quebec, Canada; 2 Division of Social and Transcultural Psychiatry, McGill University, Montréal, Quebec, Canada; 3 Cultural Consultation Service and Culturally Focused Early Psychosis Program, Jewish General Hospital, Montréal, QC, Canada; University of Alabama at Birmingham, UNITED STATES OF AMERICA

## Abstract

Clergy are often the first point of contact for religious Canadians when in mental distress, and clergy can impact help-seeking behaviours. As such, this study explores the mental health perspectives of clergy in a diverse and growing Christian denomination, the Pentecostal Assemblies of Canada (PAOC). 29 PAOC ministers with at least 2 years of experience offering pastoral care participated in semi-structured qualitative interviews, aiming to explore their mental health related beliefs, behaviours, and attitudes. Data was analyzed using thematic analysis techniques, Results indicated that participants generally held a multifactorial understanding of mental illness, including biomedical attributions and beliefs in spiritual causes and divine healing. To address mental distress, they typically offered some level of spiritual care themselves, including recommending church-based curricula. They also often urged formal service use, making referrals to mental health professionals. Notably, the sample largely reported a lack of formal mental health training and inconsistent support from the wider church. In sum, this study suggests that PAOC ministers hold nuanced views on mental health, and that clergy may require additional training and support to help them better address mental health issues in congregants.

## Introduction

The 2022 Mental Health and Access to Care Survey found that over 5 million Canadians currently meet the criteria for various mental disorders including mood disorders, social phobias, and substance use disorders [1]. The same survey revealed that only half of this population use mental health services [1], which other research has shown is often due to social and structural barriers like stigma and high costs [2–4].

Evidence suggests that such under-utilization is more pronounced among people of religious faith (particularly immigrants and minorities in Canada) who repeatedly cite a lack of religious awareness and competence among clinicians in the formal health care system [5–8]. Crucially, the significance of religion in the daily lives of many Canadians often means that they can partially or wholly impute emotional distress to spiritual causes [9,10]. This may prompt an increase in religious practices to address their distress, as well as consultation of clergy.

Research on religion and mental health indicates that *religiosity*, denoting strength of religious beliefs and engagement in religious practices such as prayer or church attendance, can indeed reduce aspects of emotional and mental distress [9–12]. This reduction in distress may occur through factors such as social support from a congregation, relief offered through prayer or consultation of scripture, and religious reframing of the purpose and meaning of suffering in a way that offers hope or encouragement [13,14].

Conversely, religious factors can also be a stressor for those with mental illness, for example by inducing guilt and shame, or alienation from their community members as a result of stigma within religious congregations and traumatic experiences in religious contexts [15,16]. For those who persist in religious participation despite such circumstances, all this can affect help-seeking behaviours, sometimes leading adherents away from medical treatments in favour of private spiritual approaches like prayer and religious healing [16–18]. Relatedly, research shows that high levels of religiosity can often lead adherents to consult clergy as a first port of call when experiencing emotional distress [19–21], however little is known about how Canadian clergy respond in such situations.

In some Christian denominations, clergy are known as pastors and their position as congregational leaders means they are highly respected [22,23]. Such pastors are typically involved in major life events of their congregants including birth (baptism), marriage (holy matrimony), and death (funeral rites). Similarly, the pastor can be a ‘go to’ person in times of distress, and are often relied upon for spiritual, psychological, and emotional help [24,25].

Consequently, pastoral attitudes, beliefs and behaviours can have a strong influence on the attitudes, beliefs and behaviours of individual congregants. This is particularly the case in the field of mental health, where clergy can help shape attitudes towards issues such as religiously inspired healing, psychiatric medication, therapy and other mental health services [26–28]. Some research indicates that clergy often embrace a holistic model when offering support to people with mental illness, stressing the importance of physical, mental, and spiritual health [29,30]. This can include spiritual approaches such as prayer for healing, the sharing of scripture, and pastoral support [21,31,32]. It can also include referral and signposting towards professional mental health clinicians and other mental health services.

Interestingly, some research from the US and UK show that clergy are typically more favourably disposed towards therapy and counselling than psychiatric medication as a recommended treatment. Indeed, some studies have shown that many clergy refer congregants to counselling or therapy or even establish counseling services in their own churches [32–34]. These positive views have been related to relevant education and various collaborative relationships between clergy and mental health service providers in local neighbourhoods [19, 35,36].

However, recent studies on clergy views of medication show more mixed results. On the one hand, one recent study of 890 clergy in the USA reported that 87% of participants would recommend prescription medication use for depression [37]. On the other hand, other US research indicates that some clergy have fears of over-prescription and distrust psychiatrists and medically qualified practitioners [38,39], sometimes leading them to counsel against use of medication [27]. Though these studies have advanced the literature, the reasons for these differing perspectives remains understudied, especially in the Canadian context.

Importantly, there is a small but growing literature on clergy’s teaching and experiences regarding mental health. One qualitative analysis of ten African-American Pentecostal pastors’ sermons showed that they actively taught that depression was a weakness [40]. Studies exploring clergy mental health and well-being report high rates of burnout and other employment-related mental distress among clergy [41–43], and these personal experiences may also shape and colour clergy responses and reactions to congregants with mental health issues. Once again, the vast majority of this research comes from the US or UK, and this is under-researched in the Canadian context.

Of note, research indicates that clergy mental health knowledge, beliefs, behaviours, attitudes and perspectives may differ according to denomination within Christianity. This typically indicates that more mainstream traditional denominations, such as Anglican or Roman Catholic, have different patterns compared to evangelical denominations [44]. One study, for example, found that Roman Catholic clergy are less likely to impute mental illness to spiritual causes than their evangelical counterparts [45]. Another more recent study found that evangelical clergy were more likely to endorse religious treatment, and less likely to encourage prescription medication use [37]. These differences further buttress the need for denomination-specific research on clergy attitudes and behaviours in Canada, especially as most previous research has focused on more mainstream Christian denominations in other countries.

One growing evangelical denomination is the Pentecostal Assemblies of Canada (PAOC), a mid-sized protestant denomination of over 235,000 members across the country [46,47]. The PAOC oversees 1,040 local congregations nationwide that operate mostly in English and French, but include congregations in languages such as Creole, Korean, Spanish, and Tamil, among others [48]. The PAOC is structurally comprised of several provincial districts that oversee local churches, and offer supports and training for pastors. Notably, the PAOC is also affiliated with the Pentecostal Assemblies of Newfoundland and Labrador (PAONL), who share the same beliefs but are governed separately.

The PAOC subscribes to specific beliefs regarding pneumatology, a theological term referring to beliefs regarding the Holy Spirit. One such belief is Spirit baptism, a belief in an internal empowering of the believer to continue the work of Christ [49,50]. Pentecostals believe that the empowerment of the Spirit enable the practice of the ‘gifts of the Spirit’ such as prophecy and healing [50,51] which have repeatedly appeared as key themes in research outside of Canada studying Pentecostal perspectives on healing [21,52]. In general, beliefs about the actions of the Holy Spirit, especially regarding healing, tend to be more intense in Pentecostal denominations in comparison with other denominations. This could have a direct impact on beliefs about mental health and appropriate responses to mental health issues, but this has been under-researched.

To our knowledge, there has been no research examining mental health related beliefs, behaviours or attitudes among PAOC clergy, even though they are often a first port of call for congregants in distress. As such, the overall aim of this study is to address this gap by documenting PAOC clergy’s mental health related beliefs, behaviours and attitudes, with a focus on (i) views regarding mental health and mental illness; (ii) their training and knowledge in mental health issues; (iii) their experiences and reactions when faced with congregants who have had mental health issues and (iv) their experiences providing pastoral care to affected individuals.

## Methods

This study employed an inductive exploratory qualitative methodology, given the lack of research on the mental health-related beliefs and practices on Pentecostal clergy in Canada. Standard procedure in such inductive studies is to enter the field with an open mind, rather than be driven by a rigid theoretical framework. In practice, this means the study is not hypothesis driven nor theory driven, but driven by a broad research question with some pre-defined domains of interest for exploration. This takes advantage of the flexibility and reflexivity inherent to qualitative research, allowing participants to use their own words, examples and explanations without having to fit their responses into a procrustean bed of researcher defined pre-existing frameworks In such studies, the goal is to document and contextualize participant perspectives, practices and experiences by adopting a stance of equipoise and neutrality, thus prioritizing bottom-up perspectives rather than researcher imposed models [53,54]. Such qualitative research is useful in unearthing the under-researched intricacies of social interactions and processes [55], warranting its use in a study of under-researched clergy knowledge, beliefs, behaviours and attitudes in the PAOC. Other work on clergy perspectives (from various religions) on mental health have also employed a similar qualitative approach [24,30].

Inclusion criteria were as follows: (i) ministerial credentials in the Pentecostal Assemblies of Canada or the Pentecostal Assemblies of Newfoundland and Labrador; (ii) experience offering pastoral care/counselling to individuals experiencing mental distress; (iii) two years of experience in ministry; (iv) ability to speak English or French; (v) able to give informed consent.

### Recruitment

The research team created electronic flyers, posters and adverts in English and French describing the study and soliciting participation, with input from two community stakeholders comprising a PAOC pastor and a congregant who agreed to assist with recruitment. JM had conversations with several individuals known to be interested in the topic while describing the nature and scope of their potential input. Upon accepting, two community members offered insight that helped the research team ensure that recruitment materials would be effective and appropriate for clergy. Furthermore, these community members were well-networked in the PAOC and one member agreed to circulate flyers through email chains and Facebook groups that were private and restricted to credential-holding clergy or PAOC staff. We also contacted officials and administrators of various provincial districts (that oversee local churches, and offer supports and training for pastors), who were asked to relay study information to their colleagues. In sum, recruitment materials circulated through online Facebook groups and posts, by researchers, collaborators, and clergy alike. Participants were compensated with $100 Amazon gift cards for their time in keeping with funding agency guidelines on the use of compensation to incentivize participation [56]. Finally, we engaged in snowball sampling, asking study participants to refer other participants in their social circles to the researchers. Recruitment took place from July 18, 2024 to August 26, 2024.

### Procedures

Interested individuals were screened to ensure adherence to inclusion criteria of the study by telephone or email correspondence. Those that passed screening participated in a single semi-structured qualitative interview over Zoom. Qualifying participants were also invited to answer a short sociodemographic questionnaire collecting basic demographic information asking simple questions such as “age” and “race/ethnicity”. In the spirit of the inductive nature of the study, race/ ethnicity was an open question allowing participants to use their own words to describe their ethno-racial background.

JM conducted all of the interviews, sometimes in tandem with AC, after receiving considerable training from the last author. This training involved attending a graduate level course on the topic taught by RW, reading papers on qualitative methodology [53–55], listening to previous interviews regarding religion and mental health conducted by the research team, and engaging in practice interviews. Participant interviews followed a topic guide constructed by the research team and reviewed by the research ethics board. This topic guide comprised of three main sections on background and ministry calling, views regarding mental health and mental illness, and experiences with congregants suffering from mental health issues. Each of these sections consisted of open-ended questions, allowing for detailed responses by the participant. In keeping with the semi-structured interview method, participants were probed for further detail and examples to clarify and contextualize their responses (54). All interviews were audio-recorded and transcribed verbatim into Word documents, and any identifying information was redacted from these transcripts, ensuring confidentiality and anonymity in the analysis. The study protocol and all study materials were approved by the Research Ethics Board of the Centre intégré universitaire de santé et de services sociaux (CIUSSS) de l’Ouest-de-l’Île-de-Montréal –Mental Health (protocol number: IUSMD 21–30) and all participants gave written informed consent.

### Participants

The initial response to outreach efforts yielded 54 interested individuals, 25 of whom were excluded as they failed to meet inclusion criteria. The final sample consisted of 29 participants whose sociodemographic information is included in Table 1. For the race/ethnicity question we collapsed responses into two broad categories (white/ non-white) in order to protect participant anonymity, as some non-white participants gave very specific responses which could reveal their participation in the study to an astute reader if race/ ethnicity was presented at a more granular level. This sample size is consistent with best practice recommendations regarding the conduct of qualitative research [57]. It permits a comprehensive understanding of participant attitudes and experiences, thus facilitating the desired ‘thick description’, while also ensuring that outlier perspectives can be elicited and documented [58]. Fifteen participants were men aged 50 and older and serve in lead pastoral roles, akin to a parish priest. Ten participants in the sample served in non-lead roles in sub-ministries such as youth ministry, missions ministry, or worship ministry, among others. Notably, five participants have completed or are enrolled in graduate studies in a mental health-related discipline and eight participants have completed mental health first-aid training. As mentioned, the PAOC consists of numerous districts across the country with varying levels of resources to offer their clergy, all of which were represented in the study, including the aforementioned Pentecostal Assemblies of Newfoundland and Labrador.

**Table 1:** Basic characteristics of participants.

Characteristic	Total Number (N = 29)
Age in years, mean (SD, range)	46.21 (12.6, 24-72)
Ministry Experience, mean (SD, range)	20.3 (11.8, 2-40)
Sex, n (%)	
*Male*	24 (82.8)
*Female*	5 (17.2)
Highest education level completed, n (%)	
*Pre-University*	3(10.3)
*Bachelor’s*	13 (44.8)
*Master’s*	7 (24.1)
*Doctorate*	6 (20.7)
Marital Status, n (%)	
*Married*	26 (89.6)
*Unmarried*	3 (10.3)
Race/Ethnicity, n (%)	
*White*	24 (82.8)
*Non-white*	5(17.2)

### Analysis

The interview transcripts were imported into MaxQDA software [59] to facilitate qualitative thematic analysis. The data analysis drew from the step-by-step thematic analysis methodology proposed by Braun & Clarke [60]. As data is being collected, this process begins with reading through transcripts and listening to sound files of recorded interviews, which JM conducted to became familiar with the data set. JM then formulated a preliminary list of codes in relation to each objective, which were edited and refined AC and RW, who also read through transcripts and listened to interviews. Upon completion of this phase, the codes were discussed by the research team and categorized into larger themes that represented common patterns, experiences and perspectives that were observed in the dataset.

### Positionality

JM is a practicing member of the PAOC though not a member of the clergy. The remaining authors are not members of the denomination, but GEJ and RW have decades of experience conducting qualitative research with a wide variety of religious people and religious communities [8,16] and used their amassed knowledge to ensure that the research team followed best practices. RW also reviewed all recruitment and interview materials prior to approval by the research ethics board. Moreover, GEJ is a medically qualified psychiatrist, AC is a medical doctor in training to become a psychiatrist, while JM and RW are social scientists. These differing individual backgrounds and experiences were strengths that contributed to a diversity of perspectives, mixing insider and outsider positions that can diminish biases associated with a homogeneous team. Indeed, such mixing of insider and outside perspectives is recommended when conducting qualitative work [61].

## Results

Interviews averaged sixty four minutes and analysis of the interview transcripts by the research team yielded three overarching themes, namely (i) a multifactorial understanding of mental illness; (ii) a holistic pastoral care approach; and (iii) a lack of training in mental health issues and mixed levels of ongoing support. All quotations are verbatim but minor changes were made to protect participant anonymity.

### Theme 1: Pastors espouse a multifactorial understanding of mental illness

This study sought to document clergy knowledge, beliefs, attitudes and explanatory models related to mental illness. This included understandings related to etiology, and understandings related to healing. When queried on these issues, most participants attributed the development of mental illness to a multitude of causes. Several participants used words like ‘holistic’ (P23) and ‘varied’ (P05) when sharing their views regarding the origin of mental health issues. For example, one participant stated that ‘it’s social. It’s spiritual. It’s biological. Sometimes it’s all three. Sometimes it’s only one of the three’ (P01). Similar statements regarding a multidimensional understanding of mental illness were found across the dataset. For example, one participant in his 40s stated:

‘We’re body, soul, spirit, right? We have different dimensions, we’re interconnected. We’re not… you can’t just separate the physical from the spiritual, or from the psychological aspect of our beings. We’re, we’re net together, and, uh, and what I put into my body, uh, all the caffeine I, I drink, or all the fatty foods I eat. I mean, you know, it affects my, my mental health. Also, who I hang out with, the stuff I watch…’ (P22).

Within this multifactorial understanding of mental illness, participants typically remarked that there was a spiritual component to mental illness. In doing so, participants sometimes referred to supernatural beings or influences including Satan, demons, or dark spirits. For example, one participant stated that, ‘the scriptures deal with, um, Jesus speaking about those who had been afflicted by demons. And so there can be a demonic influence, spiritual influence, to have a mental illness, a mental disorder’ (P18). Others drew on Christian concepts such as original sin, human imperfection, and the corruption of human nature, with one stating: ‘It’s a result of our fallen nature. I mean, we get sick in our bodies, why can’t we get sick in our minds?’(P17).

Crucially, such attributions were typically accompanied by caveats and acknowledgements of other causes and dimensions. For example, one participant in their 30’s said ‘it’s not all demons. It’s not all a person’s background either. There’s a whole, we’re complex and there’s a whole pile of spiritual issues combined with medical issues and -and mental issues… it’s a complex thing’ (P08). Indeed, several participants warned against monocausal thinking regarding mental illness whether it be solely religious explanations or solely biomedical explanations. For example, one participant in his thirties stated:

‘sometimes if we try to make it all about the devil or, or, or Satan or spirits, um, we neglect care for the people. And at the same time, if we ignore the spiritual side of it, we neglect care for the people as well’ (P06)

In fact, participants rarely identified spiritual issues as the sole cause of mental illness and some decried the exclusively spiritual understandings of mental illness that used to be predominant in the denomination. One pastor shared such a belief that used to be commonplace: ‘in the 80s, we told people if they were sick, it was because they had secret sin in their life’ (P01). Participants noted their disagreement with such views, with participant shared an anecdote portraying this disagreement: ‘I remember before hearing a pastor say when I was young, that, uh, the, some of the things in the Bible described as demon possession. It’s like, well, today we just call it schizophrenia, right?’ (P03). These views resulted in many citing a fear of ‘over-spiritualizing’ mental illness, as the same pastor shared: ‘although I see it as spiritual to some degree, I’m always very cautious to, uh, to label it in that sense for fear of, uh, fear of falling into some, some old tropes or bad generalizations I’ve seen before’ (P03). Overall, participants expressed holistic and multifactorial understandings of mental illness that included a spiritual component.

Notably, participants expressed a strong belief in divine healing and specified that and specified its importance in their theological convictions. One participant outlined this belief in religious healing, rooting it in Christian precepts: ‘we serve a God who is almighty, who can, can heal us of all of our diseases and all of our things. And that includes not just physical health. I believe it involves mental and spiritual health’ (P15). Another stated the centrality of belief in healing to denominational theology: ‘as a Pentecostal, it’s part of your doctrine that you believe that God heals’ (P03).

Prayer was considered an essential practice to facilitate such divine healing and was often imputed to belief in God’s healing abilities, as shared by this participant: ‘I try to pray with them, um, because I do believe that the Lord does heal and comfort and strengthen and help’ (P14). Such prayer was frequently integrated into church services, offering opportunity for congregants in search of healing. One pastor shared his church’s approach of weekly prayer services: ‘our altar services, part of that, we do it every week. We’re continually praying for people to be healed’ (P15).

Importantly, several participants shared anecdotes that referred to the efficacy of prayer for healing of mental health issues. For example, one shared an experience praying for a congregant who was hospitalized due to a treatment-resistant eating disorder: “so I’m dealing with a psychiatrist right now’, he says, ‘and, and he’s perplexed’… and so, I prayed with that guy that day. And he got discharged. He started eating and drinking’ (P08). Another pastor shared his experience of seeing a woman healed at a prayer service: ‘I actually know a lady who had psychosis in one of our churches. Psychosis since she was twelve, from twelve to, she was almost forty. And we saw her instantly healed and she never had a problem after that’ (P25). In sum, such personal witness and denominational theology formed the basis of belief in divine healing.

Again, participants raised several caveats regarding prayer, divine healing and related issues, noting that these spiritual factors co-existed with other factors that could aid healing. Participants typically noted that they encouraged congregants to consult clinicians, even after a perceived healing experience, and often with positive results. One pastor stated ‘if a pastor said, uh, you know, you must go see your psychiatrist, then some of them would actually listen to us’ (P26). Indeed, the dataset revealed largely positive views held by most participants regarding counseling and therapy, as highlighted by one participant: ‘therapy is good. Therapy is important. There’s no weakness in therapy’ (P01). Another participant attributed their current standing in ministry to their positive experiences with therapy, saying ‘I really believe that I’m in the game today because of my encounters, uh, with uh, professional therapists early in, early in life. And that experience, uh, I learned from that that there’s value in that’ (P22).

That said, participants expressed mixed views regarding medication, with some being favourable and some less favourable to medication. For example, one pastor shared an affirming view of medicine that was informed by his theology: ‘if sin is in the world and sin can affect a lot, including our physiology… medicine and medication can help with that’ (P13). Another noted their support for medication as part of God’s healing, stating: ‘when someone is in hospital, I go and visit them, I pray for them. I believe that God would give wisdom to doctors… so I’m not against uh, um, you know, medication’ (P29). However, others had more negative views, informed by their own interactions and experience. For example, one reflected on congregants who had taken anti-depressants, noting that ‘the symptoms from the medication have left them a lot worse, worse often than the condition’ (P15). Another stated that ‘antidepressants: I never take one myself… as I said, my friend took some of them and he killed himself’ (P07). This issue is expanded upon in theme two, where we discuss clergy approaches to spiritual care which includes signposting to services and understanding limits to solely spiritual approaches to healing.

### Theme 2: A holistic pastoral care approach

Pastoral care approaches flowed logically from the commonly held beliefs that mental illness was caused and healed by a multitude of factors, including spiritual components. Importantly, participants expressed that a central responsibility of the pastor is to offer care for those in need, as outlined by this participant’s description: ‘the word pastor means shepherd, um, you know, a shepherd, uh, cares for wounded sheep, but they also lead the main flock’ (P26). When queried on their approaches to pastoral care for congregants with mental illness, three factors were frequently mentioned: (i) individual spiritual care involving prayer and sharing of scripture; (ii) referral and signposting to local mental health services, and (iii) the use of church-based initiatives and group programs, which followed religiously inspired curricula.

Regarding spiritual care, prayer was consistently mentioned as an extremely important element. This included the pastor praying with participants, for participants, as well as encouraging them to pray alone. For example, one participant stated ‘I guess, [I] offer them spiritual care as much as I can. Pray with them, pray for them. Uh, reassure them of the promises of God, his faithfulness, his goodness’ (P03). Another participant described a similar approach to spiritual care, describing it as a key vocational responsibility: ‘it begins and ends with prayer. And my role is to remind them of what this, what the scriptures actually say. It’s my job’ (P17).

Indeed, referring congregants to scripture was also considered an important aspect of spiritual care, as this was considered to provide solace and support. This included referral to the Psalms, the healing stories of Jesus in the gospels, or the encouragements in the Book of Romans. For example, one participant stated ‘there’s enough examples in the Bible for us to, I think, find solace. I think the psalmist, uh, is -is a great place. 150 Psalms for all seasons of life’ (P09). Another pastor shared a more general example, sharing ‘scriptures about like how like God delights in like showing mercy over judgment and like God is compassionate and like slow to anger’ (P23).

Interestingly, participants recognized their own limits when engaging in spiritual care for people with mental health issues and sometimes set boundaries to prevent issues or misunderstandings. For example, some participants sometimes expressed discomfort in offering such care to congregants of the opposite sex and had several measures in place to mitigate potential issues such as involving their spouse, referring to another same sex staff member, or involving another staff member in any support sessions. For example one female participant stated ‘the preference that we both, my lead pastor and I go with… is that we try not to counsel the opposite sex if avoidable (P23)., while another refused to counsel females with mental health issues citing: ‘personal things…I just don’t think it’s healthy for me to be dealing with those type of things with a female, there can be attachments that can take place’ (P21). This behaviour is consistent with safeguarding guidelines widely used by many religious organizations to prevent potential exploitation and abuse of congregants.

Another limit involved time and duration. Most pastors offered individualized support sessions for congregants in mental distress but typically set a limit of around three to six sessions of one hour each. One pastor noted this was sufficient to communicate useful skills and support, stating: ‘any tool that I have that’s helpful and beneficial for you in a spiritual capacity or the pastoral capacity… by six sessions, you have every tool that I have to give you’ (P01). Another similarly noted that issues still arising after a handful of sessions would better be treated by a mental health professional, stating that ‘after, like, three to six counseling sessions, if we’re like, oh...they have deep needs, we would then refer them to a psychotherapist’ (P23).

Indeed, as mentioned in theme one, referral to mental health professionals was another frequent approach, with participants recognizing their own limits as well as the added value of having counselors and therapists involved in care. For example, one pastor stressed the danger of counseling congregants beyond their training: ‘pastors aren’t trained to be counselors and therapists, um, and we need to be aware of just kind of how much damage we can do, uh, if we don’t have those skills and, and training’ (P24). Another pastor succinctly stated: ‘there comes a point where a sheep needs a vet, you know’ (P26), using the previously cited shepherd metaphor to highlight the need for professional mental health care, in addition to any support offered by clergy. Interestingly, participants mentioned a preference for referring to Christian professionals, often due to the aforementioned belief in the spiritual component of mental health, as well as better alignment in core values. One pastor explained this preference: ‘I’m most comfortable referring them to a Christian counsellor. Because I’m, I’m viewing it as, spiritual in nature, right? (P16)’.

The third and final method employed by pastors to address congregational mental health issues was church-based mental health provision, and group programs following religiously inspired curricula. Interestingly, participants discussed several types of church-based initiatives that helped congregants obtain access to counseling. For example, one stated that: ‘we’ll pay for most of the counseling. I say 90 percent because our people can’t afford it and people give us deals…we put money in our budget for that’ (P05). Others mentioned taking on counsellors as temporary church staff to help with people dealing with difficult situations, with one stating that: ‘we even had someone… kind of working very part-time or contract-basis doing some kind of emergency counseling with people at our church to help them through different situations’ (P24). Beyond these initiatives, some PAOC churches have built larger-scale counseling centers in collaboration with their local governments to offer low-cost services to the wider community. One pastor shared the result of this collaborative relationship: ‘we’re a partner with the regional municipality… we get a lot of our clientele who just walk off the street. But we offer free services for, uh -first three sessions is free: no cost whatsoever’ (P22).

Additionally, several participants noted that their churches had set up group programs that followed religiously inspired curricula to help congregants with mental health issues. These typically ran midweek in the evening in local churches, and were led by a facilitator that often was not the pastor. Participants cited a diversity of programs with common elements. Many focused on addictions, for example some participants noted that they offered the Christian 12- step *Pathway to Peace.* Others implemented *Celebrate Recovery*, a Christian addiction recovery curriculum, while others offered *The Genesis Process*, a neuroscientifically-informed Christian holistic health curriculum. Participants spoke favourably about these programs, describing them as ‘powerful’ (P21), and ‘successful’ (P14).

Several participants mentioned Neil T. Anderson’s *Steps to Freedom in Christ*, a series of theological affirmations and prayers meant to set people free from ‘demonic activity’ in their life. One participant who often recommended this program to congregants with addiction described its basic structure: ‘a person would go and they would, they would confess, uh, things that had happened in their lives and they would confess that sin and they would hand that over to God in the process’ (P15). As can be seen from the concepts used (e.g., ‘confess’, ‘sin’, ‘demonic activity’), the underlying healing framework of these programs was typically grounded in theology, rather than mainstream psychology or psychiatry.

In sum, participants typically took a multi-pronged approach to spiritual care for people with mental health issues, comprising individualized supports involving prayer and scripture, referral and signposting to counsellors and therapists, and provision of religious and non-religious programming in the church context.

### Theme 3: A lack of training in mental health issues and mixed levels of ongoing support

Training in mental health issues, or lack thereof, was frequently mentioned by participants. Indeed, most participants expressed low levels of preparedness to deal with mental health issues in congregants due to a lack of training, an experience succinctly articulated by one participant: ‘we’re not trained to handle... the mental health challenges that we encountered in ministry’ (P02). Another pastor shared that his district offered coaching training to equip pastors, but that this was unsuited to congregational needs: ‘Um, I don’t find life coaching, uh, really translates into pastoral counseling very well, though. Um, it is more aimed at um, you know, helping people who are successful at life be a little more successful’ (P26).

Similarly, other participants noted that ministerial training left out important practical issues related to human interactions and inter-personal psychology, with one senior pastor stating: ‘they might teach you how to do a board meeting, but they don’t teach you about the aggressive board member…they didn’t teach you how to practically, um, use your theological concepts’ (P06). This lack of training often left participants having to learn in real time to meet the messy everyday human demands of congregants, as highlighted by one pastor: ‘between weddings and funerals, there seemed to be a vacuum of, uh, you know, just trust God, open the Bible and see what happens. You know, that’s basically all you got’ (P09).

Interestingly, many participants took active steps to fill this vacuum. As mentioned earlier, some even completed formal education in counseling or mental-health disciplines to this end. One children’s pastor shared his experience seeking certification: ‘I personally have taken the initiative to, uh, go to a couple of behavioural therapy courses. Because I deal with kids particularly, so what I’m coming across a lot of is behavioural issues’ (P01). Another pastor sought trauma-informed care training and used his education to establish a mental health team in his church: ‘we have a lot of people in the medical field in the church. So one day I gathered everyone and I said, Hey, um, I’d like to put together a mental health team so that whenever the pastoral team meets with people, if we see that, you know, uh, what they require is, is something more specialized in terms of, care, then I’d like you guys to be the go to’ (P12).Notably, eight pastors completed Mental Health First Aid training as part of their Bible college training and some even subsidize this training for congregants to encourage peer support within their congregations.

Cognizant of the limits expressed in theme two, participants mentioned alternative supports that could help them to address congregational mental health needs effectively. Crucially, our data suggest that the success of this endeavour typically varied according to location, with urban participants reporting a myriad of available resources and supports, while rural participants noted a paucity. Indeed, pastors of churches in urban and higher income areas reported consistent and reliable access to many local mental health resources. For example, one urban pastor shared that she would easily enlist the help of local crisis outreach and support teams services in her area. Another pastor cited his use of Victim Services, who could help members of his church who experience harm. Other pastors have access to long lists of local mental health professionals and support organizations, with one pastor of an urban congregation of over 3000 members stating: ‘in my office, you will see, um, we have all the emergency numbers there, mental health and all of that…. so we, as a church, we are very, very connected’ (P11).

In contrast, pastors of rural churches with lower levels of membership reported access to fewer resources. One pastor of a rural church shared their struggle to obtain services for congregants: ‘I think we have a clinician who comes maybe once a month or something to our town, or once every two weeks’ (P27). A youth pastor shared similar concerns, emphasizing the lack of school counselors for children in the church exhibiting behavioural issues: ‘in this rural isolated area, there are four or six schools that are sharing one counselor... so there’s, you know, no help for any of these kids’ (P04). The shortage of services in rural communities have prompted some churches to fill the gap and offer care for people with mental illness in their communities, supplying them basic necessities in addition to spiritual care. For example, one pastor noted that her church was a front-line provider of essential supports to people with more serious mental illness including provision of food and clothes, stating: ‘nobody with the, like that level of, of serious mental illness, um, can stay here long cause there’s just nothing for it. There’s no support at all… we end up involved in those kinds of things because there are no resources’ (P27).

Much like local resources, participants expressed variable levels of resource support offered by their provincial districts and their district leaders. Some described their districts as ‘supportive’ (P11) and‘progressive’ (P01) in the area of mental health supports. Crucially, these districts were typically larger in size and had more substantial budgets. Several participants highlighted one wealthy district’s emphasis on mental health and its support of pastors, citing positive experiences attending their mental health training seminars. Similarly, one participant highlighted the ongoing interest of their district superior in mental health, including ministers’ own mental health, stating: [they’re] in touch with me on a monthly basis, pretty much every two months, just how am I doing? How’s my family doing? How am I doing? How’s my mental health? How’s my spiritual health?’ (P08). Indeed, when discussing supports regarding mental health, participants frequently took the conversation towards their own personal experience when faced with their own mental health issues, using this as a lens to reflect upon wider attitudes towards mental health by church leadership and church bureaucracy.

In this regard, the majority of participants reported negative experiences with support offered by their provincial districts. One pastor shared how he used district services for burnout: ‘so I was like, great. I can talk to somebody that gets what I’m going through kind of thing. And I remember coming back, um, to my wife after the meeting saying the guy talked about himself the entire time’ (P20). Another pastor shared that an important gap in his own search for services, saying ‘our district has had an attitude of like, you know, here’s, here’s 500 bucks, here’s a list of counselors, go sort it out on your own’ (P27).

Though every provincial district offers subsidized counseling for clergy in need of support, several participants reported a hesitancy to use these supports, often due to fears of breaches in confidentiality or of negative repercussions. For example, one pastor shared an experience of seeking help from denominational supports: ‘…you have to go talk to somebody to get a number, to get approval, which like everybody, everybody’s going to know’ (P10). Another pastor shared similar fears, sharing a commonly held reluctance to disclose personal issues to district supports:

‘do you go to the person who, who could cut off your credentials and say, hey, I’m really, …struggling with my thought life…who do you go to? It’s like going to your boss and saying, you know, yeah, boss, I’ve been stealing money from the accounts and can I take some time off and get to the root of it? …so that’s kind of how a pastor feels when the district offers them things’ (P21).

Given these support-related issues, most participants relied on peer support as an alternative, as highlighted by one lead pastor: ‘it’s one of the best decisions I’ve made since I’ve become a pastor to surround myself with people… we talk about everything, we pray for each other, and then we go through stuff together’ (P12). For others, this involved reaching out to board members of the church and other church leaders for support. Finally, several pastors mentioned their spouses as being key sources of support, with one stating ‘My marriage has been strong. My relationship with my kids has been strong. And so that has, has always been a pretty big saving grace for me is when I get home, things are quite healthy’ (P19).

## Discussion

This study has three key findings. First, mental illness was typically attributed to a variety of causes including biological, social, and spiritual factors. This multifactorial understanding expressed by study participants is consistent with older research showing similar understandings among clergy outside Canada and from other denominations [29,30]. This study shows a strong opposition to spiritual factors as monocausal explanations of mental illness but overlaps with other research that illustrates that mental health is understood, in part, spiritually by the clergy [62,63].. Interestingly, numerous participants highlighted the evolution of the denomination from one which perceived mental illness through a solely spiritual lens, to one that now incorporates multi-factorial understandings. Various factors could account for this, including increased exposure and general awareness of mental health across Canadian society. Moreover, older participants mentioned an absence of mental health-related discussion in Bible college, while newer graduates mentioned the inclusion of Mental Health First Aid (MHFA) training, which has been shown to increase mental health literacy [64,65].

Participants typically stated a strong belief in the efficacy of divine healing for mental illness, though not as a sole and exclusive method of healing. Other studies have reported such beliefs among clergy, but these data are from outside Canada and have typically focused on mainstream denominations [31,66]. As previously mentioned, divine healing is a staple of Pentecostal theology and is a key component of PAOC beliefs as expressed in their doctrinal treatise [49]. These beliefs were also shown to have been reinforced by personal experiences with many participants sharing stories of witnessing healing as a result of prayer. Reports of such divine healing is also a well-documented phenomenon in global research of Pentecostals [21,52], but these studies do not document prayer for divine healing as a tool for clerics to address mental illness within a wider range of approaches. Participants did note that many who sought divine healing did not become completely healed, leading participants to recommend conventional treatments such as therapy, counselling and medication. In other words, participants recognized and recommended diverse modalities of healing and recovery, depending on the individual and the situation.

This leads to the second key finding, which is that is participants took a variety of approaches to aid congregants with mental health issues, including one-on-one pastoral care, referral to official mental health services, and the provision of faith-based group programs. Pastoral care approaches consisted primarily of a series of one-on-one meetings focused on healing prayer, reference to scriptures and other religiously inspired counsel. All of these have been repeatedly witnessed in other research exploring clergy actions when faced with congregants with mental illness, although there is still a need to demonstrate the efficacy of these methods [21,32,62]. Indeed, these recommendations have influenced approaches to addressing mental illness by congregants of various religions [16]. This study found a strong preference for same-sex pastoral counseling, which is a common practice in Christian counseling [67].

Interestingly, participants typically expressed strong support for the utilization of therapy and counsellors by their congregants in mental distress. The results are replete with instances where participants spoke highly of these interventions, and there are many examples where participants referred congregants to these services. These positive views of counseling are consistent with research showing that the majority of clergy refer congregants to professionals [34], but again these findings are from outside Canada and are not specific to Pentecostals. This affirms the hypothesis that denominational differences can impact pastoral approaches, as studies of clergy belonging to other denominations have often cited mixed views of counseling.

That said, participants expressed more equivocal views regarding psychiatric medication. These concerns centred on various issues. On the one hand, some participants indicated a strong belief in the efficacy and utility of medication, and related their beliefs regarding medication to their theological perspectives. On the other hand othersexpressed worries and concerns about medication, mainly in relation to potentially harmful side-effects ornegative personal experiences regarding medication. Interestingly, these equivocal views have been shown in other research on other denominations, where fears of over-prescription tended to be the most common underlying reason for dissuasion of medication usage [38,39]. One American survey of 890 clergy from various mainstream denominations (mentioned in the introduction) found that 87% of participants would encourage the use of prescribed medication for congregants with mental illness [37]. All this indicates a diversity of attitudes within Christian denominations, with Canadian Pentecostal ministers perhaps veering towards more sceptical views of psychiatric medication, especially as a sole route to recovery [45]. This may be due to the previously discussed preference for spiritual explanations typically associated with evangelical denominations.

Consistent with doubts about the efficacy of mainstream treatment approaches, several participants cited their use of faith-based curricula (often in group settings) that address issues related to mental illness. Specifically, four curricula were often mentioned by participants, however there is a lack of empirical research on the efficacy of these approaches. To our knowledge, only *Celebrate Recovery* has a corpus of research literature supporting its usefulness [68,69]. Importantly, half of the mentioned programs are newer, which may mean that they have not yet been formally evaluated. While these programs were generally positively appraised by participants, there is an urgent need for research on these programs to document efficacy, effectiveness, and utilization. This could include a stratified approach that controls for variables such as age, sex, immigrant status, and religious denomination. Of note, research of similar 12-step programs in religious populations have yielded mixed results [70,71], further indicating the need for more research on these programs.

The third key finding is that participants reported a lack of training in pastoral care for people with mental illness, and mixed levels of ongoing institutional support. Inadequate training in mental health is consistent with other research conducted on non-Canadian and non-Pentecostal samples [72,73]. As previously discussed, lack of training left clergy feeling unsure about their skills, with the result that they often appropriately referred distressed members of their congregations to mental health professionals. Notably, successful referrals were shown to be highly contingent on access to resources made available to clergy, with pastors of rural churches having less access and reporting more difficulty to find help for congregants. This reflects more general research on the lack of mental health and social care resources available to Canadians in rural areas [74]. Relatedly, participants expressed mixed levels of support from the provincial districts that support pastors of churches, with many noting an urgent need for increased supports. Participant efforts to address this lack of training involved peer support and self-initiated efforts to increase mental health literacy, both of which have been shown to improve mental health [75–77].

### Limitations

There are a number of limitations to this study. To begin, the sample consisted primarily of white, Canadian-born, Anglophone men, which is reflective of the wider population of PAOC Ministers,. However as mentioned in the introduction, the PAOC has congregations that operate in numerous languages, often led by pastors from diverse ethno-racial backgrounds. Understanding the experience of ministers under-represented in the present study, for example women and ethnic minority pastors serving immigrant communities, should be an area for future research, as they may have different experiences or attitudes. Secondly, we employed a ‘lumping’ approach, meaning that the analysis was not stratified by a number of potentially important variables such as age. For example, younger participants may have been exposed to mental health messaging during their education at Bible college and elsewhere. As such, stratifying by age may have led to differing results, which would have implications for clergy-led mental health interventions. Relatedly, pastors in the PAOC can hold different levels of pastoral credentials, each with their own levels of required training. Stratification by credential level may have also revealed important training-related differences. Thirdly, this study carried the risk of researcher bias, given JM’s membership in the denomination, meaning that he may have been inclined to paint a positive picture of the denomination to which he belongs. However, several steps were taken to minimize this risk, as discussed in the methods section, including protocolized interviews, teamwork, close supervision, and multiple coding of the raw data. Fourthly, this research may have been influenced by sampling bias inasmuch as the recruitment techniques could have disproportionately favoured participation of pastors interested in mental health, which, in turn, negatively impacts generalizability to the larger PAOC clergy. This is a real possibility given the lack of ethnic diversity among the study participants.

### Practical implications and future research

The findings of this study point to various courses of action. Firstly, while participants were conversant with mental health issues and demonstrated awareness of impacts on congregants, professed low levels of preparedness to address mental health needs suggest the need for greater faith-based trainings in this area. This could involve mental health literacy initiatives like lectures or seminars at Bible colleges and training institutions or self-directed online courses with educational content for clergy members. This may also involve reference to faith-based pastoral counseling manuals [78,79]. Perhaps the PAOC could implement a certification program for its pastors to ensure a minimum level of mental health competency in addition to a support network where difficult cases could be confidentially reviewed by peers.

Relatedly, this study revealed that participants had concerns about stigma and confidentiality when they experience their own mental health issues. One potential solution may be the outsourcing of counseling services from providers with adequate distance from the denomination but who share theological beliefs, much like healthcare workers who obtain services from professionals outside their immediate networks. Another need for clergy, especially those in areas with poor access to mental health resources, are lists of service providers. This is a common issue in large countries with sparse and spread-out populations outside major cities, such as Canada and Australia and parts of the United States. Interestingly, these countries have implemented considerable telehealth programs as a consequence of COVID and recent technological advances [80]. These can be useful for rural communities where options for service providers are limited.

Secondly, this research highlights the need for greater collaboration between clergy and the professional mental health community in Canada. Participants from larger, more well-resourced districts were grateful for mental health worker supports, but the lack of mental health services highlighted by other pastors is an important concern. This gap may be addressed by the organization of networking events, allowing clergy to connect with mental health service providers in their areas to foster mutually beneficial relationships. Additionally, existing research shows the importance of religious awareness and competence in clinical practice, a gap that clergy might be able to fill [8]. One example of such a collaboration is the Clergy Outreach and Professional Engagement (COPE) model, with clergy supporting clinicians in equipping clients with religious coping strategies and eventual integration into religious communities [35]. Another approach may be to replicate those from outside of the religious domain, where Canadian researchers have collaborated with community stakeholders to produce mental health educational videos that are tailored to specific contexts [81].

Thirdly, this study has opened a multitude of potential avenues of research in mental health and religion. At a wider level, this study raises the need to further identify specific gaps in mental health literacy at the clergy level and an assessment of potential solutions to knowledge gaps. This could expand to an evaluation of the efficacy of interventions used in church settings such as the aforementioned newer faith-based curricula. Relatedly, the importance of district-level supports highlighted in this study suggest that further district-specific research may be beneficial. Notably, numerous denominations in multiple religions have similar hierarchical structures, and implementation of this study’s findings in different faith groups could improve existing knowledge of the relationship between religion and mental health.

## Conclusion

In sum, this study reveals that PAOC clergy hold a multifactorial model of mental illness including spiritual beliefs like divine healing. This model often prompts PAOC clergy to employ prayer for healing when encountering congregants with mental illness. Participants also engage in pastoral care when working with individuals with mental illness, a practice mainly consisting of scriptural teaching and prayer, referral to official mental health services, and provision of church-based programs following faith-based curricula to encourage congregants and relieve distress. Referral practices include church subsidies for struggling congregants and in-house counseling services. Church curricula were affirmatively discussed, but further research is needed to assess the content and efficacy of such programs. Finally, participants frequently shared a belief that pastoral training failed to equip them to meet congregational mental health needs, often prompting them to rely on peer support or autodidactic mental health training. All this indicates the need for more education and training of Pentecostal Canadian clergy, as well as concomitant research evaluating the efficacy of new and existing programs.
